# Maternal High Fat Diet Alters Gut Microbiota of Offspring and Exacerbates DSS-Induced Colitis in Adulthood

**DOI:** 10.3389/fimmu.2018.02608

**Published:** 2018-11-13

**Authors:** Runxiang Xie, Yue Sun, Jingyi Wu, Shumin Huang, Ge Jin, Zixuan Guo, Yujie Zhang, Tianyu Liu, Xiang Liu, Xiaocang Cao, Bangmao Wang, Hailong Cao

**Affiliations:** ^1^Department of Gastroenterology and Hepatology, General Hospital, Tianjin Medical University, Tianjin, China; ^2^Department of Pathology, General Hospital, Tianjin Medical University, Tianjin, China

**Keywords:** maternal high fat diet, offspring, intestinal development, microbiota, colitis

## Abstract

**Background:** Accumulating evidence shows that high fat diet is closely associated with inflammatory bowel disease. However, the effects and underlying mechanisms of maternal high fat diet (MHFD) on the susceptibility of offspring to colitis in adulthood lacks confirmation.

**Methods:** C57BL/6 pregnant mice were given either a high fat (60 E% fat, MHFD group) or control diet [10 E% fat, maternal control diet (MCD) group] during gestation and lactation. The intestinal development, mucosal barrier function, microbiota, and mucosal inflammation of 3-week old offspring were assessed. After weaning all mice were fed a control diet until 8 weeks of age when the microbiota was analyzed. Offspring were also treated with 2% DSS solution for 5 days and the severity of colitis was assessed.

**Results:** The offspring in MHFD group were significantly heavier than those in MCD group only at 2–4 weeks of age, while no differences were found in the body weight between two groups at other measured time points. Compared with MCD group, MHFD significantly inhibited intestinal development and disrupted barrier function in 3-week old offspring. Although H&E staining showed no obvious microscopic inflammation in both groups of 3-week old offspring, increased production of inflammatory cytokines indicated low-grade inflammation was induced in MHFD group. Moreover, fecal analysis of the 3-week old offspring indicated that the microbiota compositions and diversity were significantly changed in MHFD group. Interestingly after 5 weeks consumption of control diet in both groups, the microbiota composition of offspring in MHFD group was still different from that in MCD group, although the bacterial diversity was partly recovered at 8 weeks of age. Finally, after DSS treatment in 8-week old offspring, MHFD significantly exacerbated the severity of colitis and increased the production of proinflammatory cytokine.

**Conclusions:** Our data reveal that MHFD in early life can inhibit intestinal development, induce dysbiosis and low-grade inflammation and lead to the disruption of intestinal mucosal barrier in offspring, and enhance DSS-induced colitis in adulthood.

## Introduction

The incidence and prevalence of IBD, a chronic immunologically mediated intestinal disorder including ulcerative colitis (UC) and Crohn's disease (CD), has been increasing worldwide, especially in Asia (1–3). This epidemiological evolution has been paralleled with westernized diets and lifestyles (4). The pathogenesis of IBD remains unknown. Growing evidence reveals the development of IBD is associated with host genetic susceptibility (5), environmental risk factors (6), and gut dysbiosis (7, 8).

Lately early life events including delivery mode, breastfeeding, maternal diet, and antibiotics use are recognized as potential risk factors for IBD (6, 9, 10). Although recent studies suggest that the intrauterine environment and placenta is not sterile as once presumed, the initiation period of neonate microbiome colonization remains obscure (11–13). Particularly in early life, the composition and diversity of the colonized microbiota profoundly influence the development of host immune system and maintain host health in later life (14, 15). Recent studies have elucidated that several factors including prenatal environment, delivery mode, and postnatal factors can shape the microbial communities of the neonate at birth and later (16). Maternal microbiota, mainly including gastrointestinal microbiota and vaginal microbiota, and breast milk composition play an essential role in the colonization of neonate microbiome (17).

It is commonly accepted that maternal diet is one of the major factors influencing offspring microbial composition (18–20). Interestingly, regulation of maternal microbiota composition via supplementation with probiotics or prebiotics in early life has been shown to provide beneficial effects for neonatal gastrointestinal tract (14, 21, 22). In contrast, both epidemiological evidence and experimental data suggest that maternal high fat diet (MHFD) during gestation and lactation could lead to colonization of specific pathogenic bacteria and further increase the risk of diseases of progeny (23–26). A recent study has reported maternal high fat diet consumption can enhance the susceptibility of offspring to Dextran sulfate sodium (DSS)-induced colitis (27). However, the underlying mechanisms and the role of microbiota have not been defined. Given the potential influence of MHFD on offspring, we hypothesized that MHFD could induce gut microbiota perturbation and thus exacerbate the susceptibility of offspring to colitis in adulthood.

In the present study, our results showed that MHFD altered intestinal development and cell proliferation, induced dysbiosis and low-grade inflammation and disrupted mucosal barrier function in offspring. More importantly, MHFD significantly altered the composition of offspring intestinal microbiota and further enhanced the susceptibility to DSS-induced colitis in adulthood. These findings suggest that MHFD in early life can negatively impact intestinal development and function, as well as cause a marked shift in gut microbiota, and then predispose the offspring to facilitate the development of colitis. Therefore, this study opens the possibility of understanding the pathogenesis of IBD in adulthood induced by MHFD in animal models.

## Results

### MHFD inhibited the intestinal development of offspring in early life

**Figure 1:**
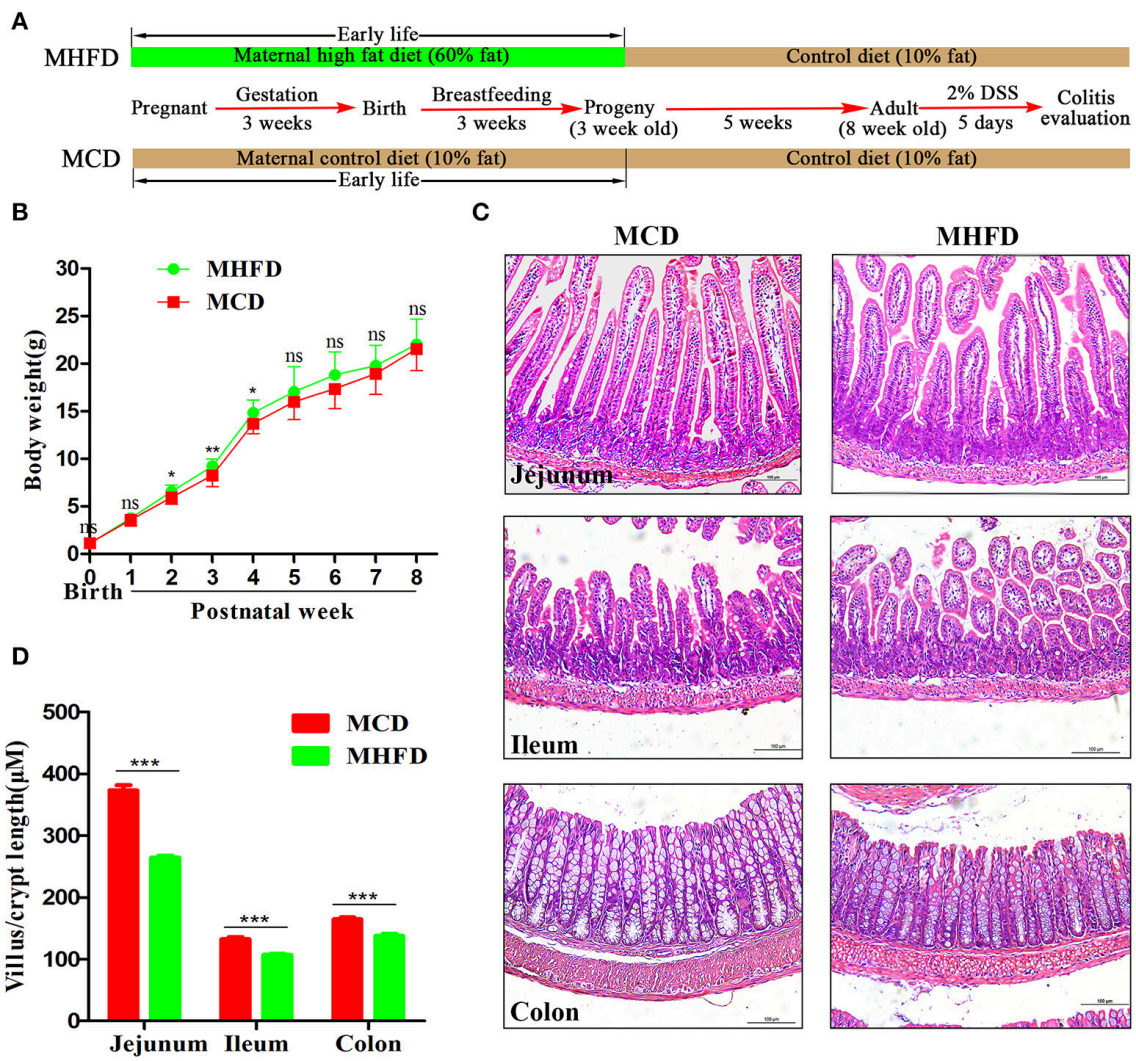
Maternal high fat diet altered the intestinal development of 3-week old offspring mice. Maternal and offspring mice were treated as described in **(A)**. Bodyweight of pups was recorded at birth and weekly until 8 weeks **(B)**. Intestinal and colonic tissues from 3-week old mice were performed by H&E staining **(C)**. The length of villus and depth of crypt were measured in at least 100 villi and crypts per mouse **(D)**. MHFD, maternal high fat diet. MCD, maternal control diet. DSS, dextran sulfate sodium. In **(B–D)**, MHFD: *n* = 20, MCD: *n* = 15. Scale bar: 100 μm. ^*^p < 0.05, ^**^p < 0.01, ^***^p < 0.001.

Previous studies have demonstrated that maternal obesity or high fat diet may contribute to offspring obesity (28–30). In this study, we indeed found that the offspring in MHFD group had slightly increased body weight at 2–4 weeks of age, with no significant difference at other measured time points (Figure 1B). Given maternal diet is the only variable factor for the pups before 3 weeks of age, 3-week-old as a key turning point was chosen for evaluating the effect of MHFD.

The intestinal villi play an important role in nutrient absorption due to increasing the surface area of absorption. As villi length is commonly accepted for evaluation of intestinal growth (31), we examined morphological expressions to determine the intestinal growth of offspring in both groups. H&E staining showed the length of villi and depth of crypts of 3-week old offspring in MHFD group were significantly decreased compared to those in MCD group (Figures 1C,D). Similar findings were not seen at 8 weeks (data not shown).

**Figure 2:**
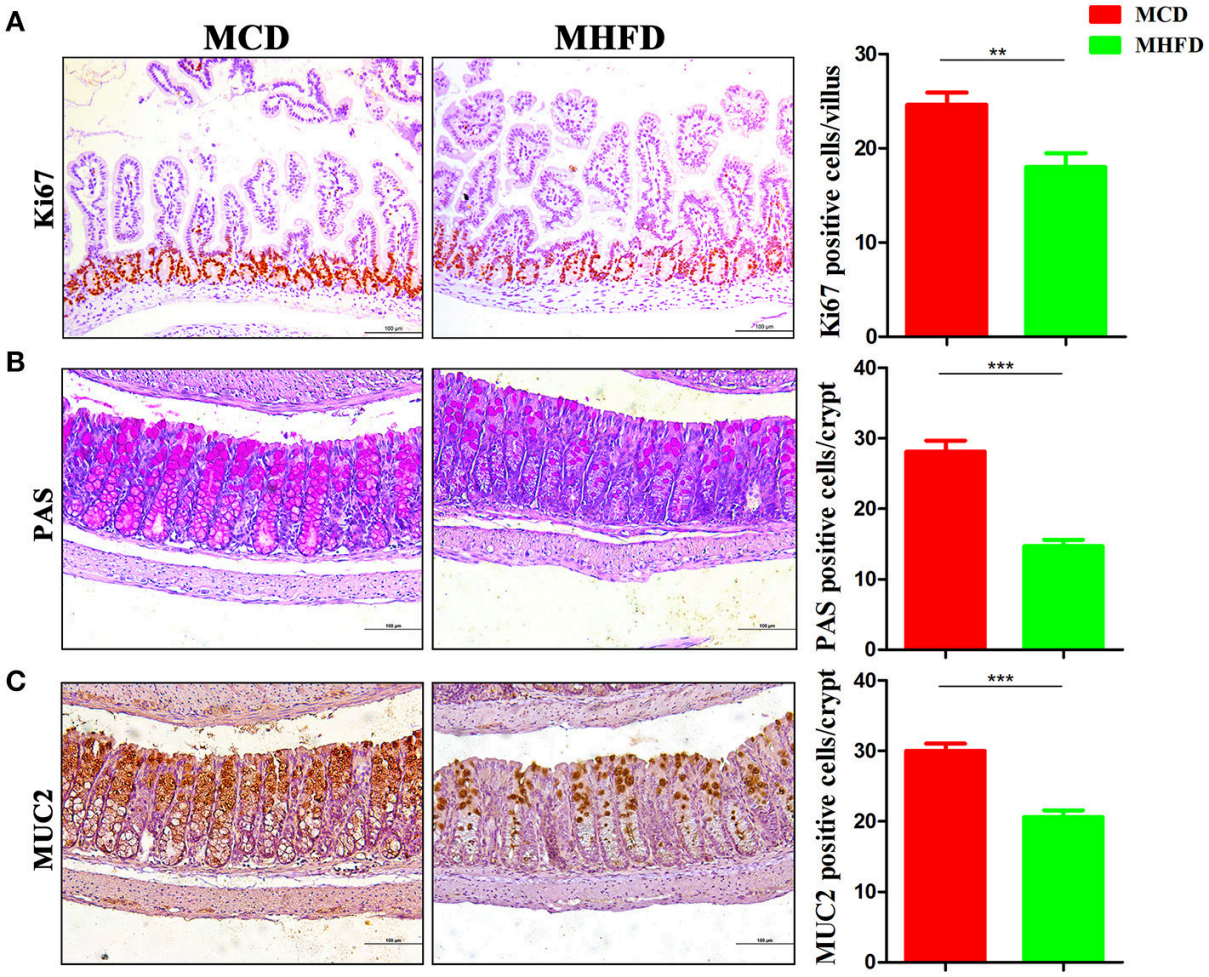
Maternal high fat diet inhibited intestinal proliferation and differentiation of 3-week old offspring mice. Proliferation (Ki67) in the small intestine was assessed by immunostaining **(A)**. Goblet cells in the colon were assessed by Periodic acid Schiff staining **(B)** and MUC2 in the colon was assessed by immunostaining **(C)**. The numbers of positively stained cells in each villus/crypt were shown. MHFD, maternal high fat diet. MCD, maternal control diet. In **(A–C)**, *n* = 6 for each group. Scale bar: 100 μm. ^**^p < 0.01, ^***^p < 0.001.

The development of the gastrointestinal tract was mainly modified by maternal nutrition until the third postnatal week in rodents (32, 33). Epithelial cells in the villi are renewed through cell proliferation, differentiation, and migration period. Various mature intestinal cells differentiate from multipotent stem cells located in the intestinal crypts (34). Here we investigated the effect of maternal diet on intestinal cell proliferation and differentiation of 3-week old mice. Ki-67 staining showed decreased proliferating cells in pups of MHFD group (Figure 2A). In parallel, the number of goblet cells (as indicated by PAS staining) and MUC2 positive cells in each crypt which indicate differentiated cells were significantly decreased in MHFD pups (Figures 2B,C). Meanwhile, the expression of MUC2 gene in the colon was also decreased in MHFD group, as compared to the offspring in MCD group (Supplementary Figure 1). Together these findings indicate that MHFD can alter the intestinal development and cellular differentiation of progeny in early life.

### MHFD altered composition and diversity of gut microbiota in 3-week old offspring mice

**Figure 3:**
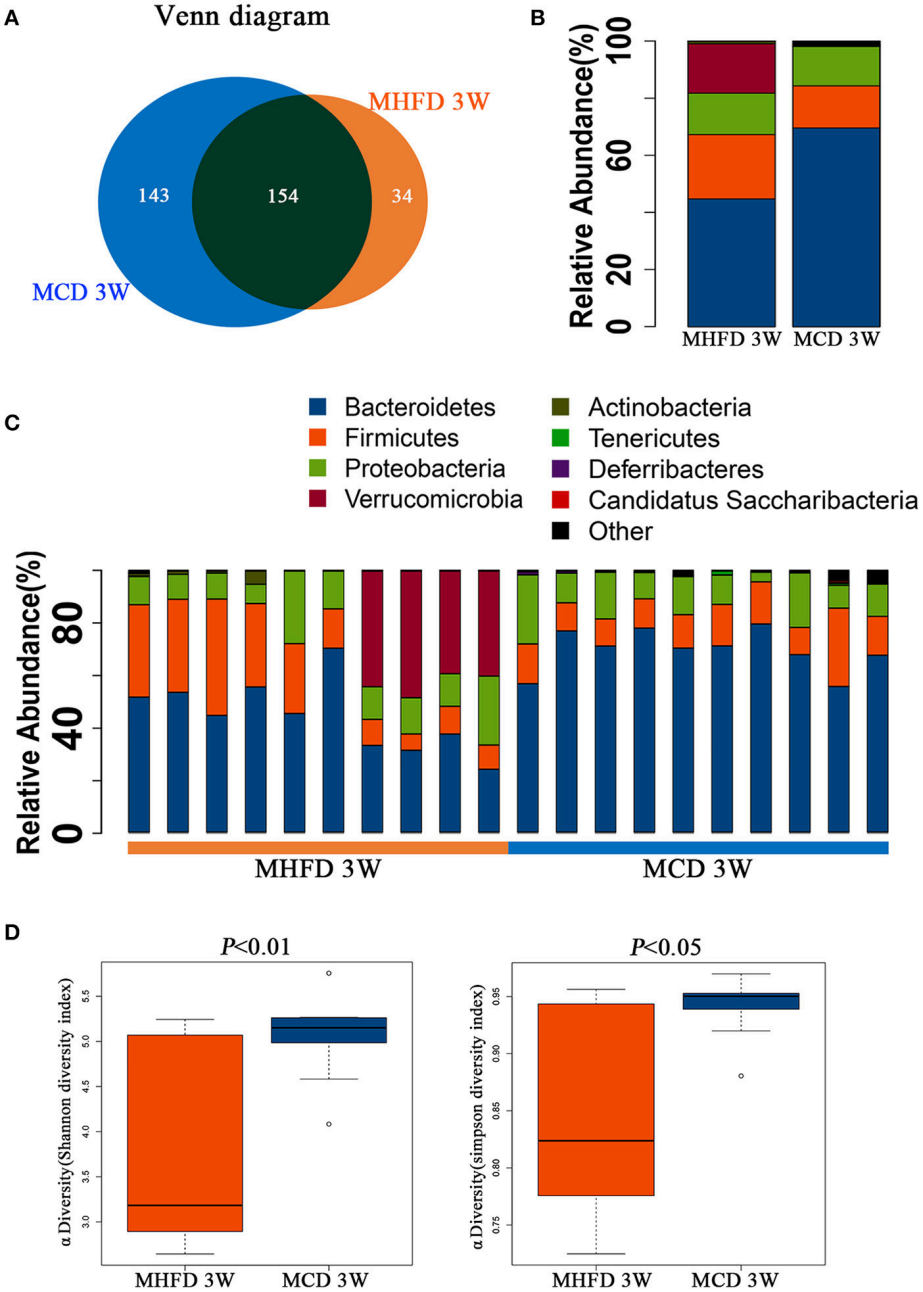
Maternal high fat diet altered the composition and α diversity of gut microbiota in 3-week old offspring mice. Total fecal bacteria from each 3-week old offspring mice were detected by 16S rRNA sequencing. Venn diagram **(A)**, relative abundance of bacterial taxa at the phylum level between two groups **(B)** and in each mouse **(C)**, Shannon and Simpson diversity index **(D)** was shown. MHFD, maternal high fat diet. MCD, maternal control diet. In **(A–D)**, *n* = 10 in each group.

We utilized 16S rDNA sequencing to characterize and quantify the gut microbiota of 3-week old offspring. We evaluated OTUs of MHFD group and MCD group to identify the unique and shared species. Venn diagram showed there were 188 OTUs in MHFD group and 297 OTUs in MCD group, with 154 OTUs shared (Figure 3A).

Next we analyzed the microbiota community structure of each mouse in MHFD and MCD group. Four dominant phyla, which are *Firmicutes, Bacteroidetes, Proteobacteria*, and *Verrucomicrobia*, were evaluated in order to compare the microbial differences between two groups at the phylum level. Notably, in the MHFD group, the relative abundance of *Firmicutes* and *Verrucomicrobia* were elevated with the reduction of *Bacteroidetes*, and the *Firmicutes*/*Bacteroidetes* ratio was increased simultaneously (Figure 3B). The microbial composition at the phylum level in each mouse was shown in Figure 3C. The Shannon and Simpson diversity index revealed that MHFD significantly decreased the alpha diversity of 3-week old offspring microbiota (Figure 3D).

**Figure 4:**
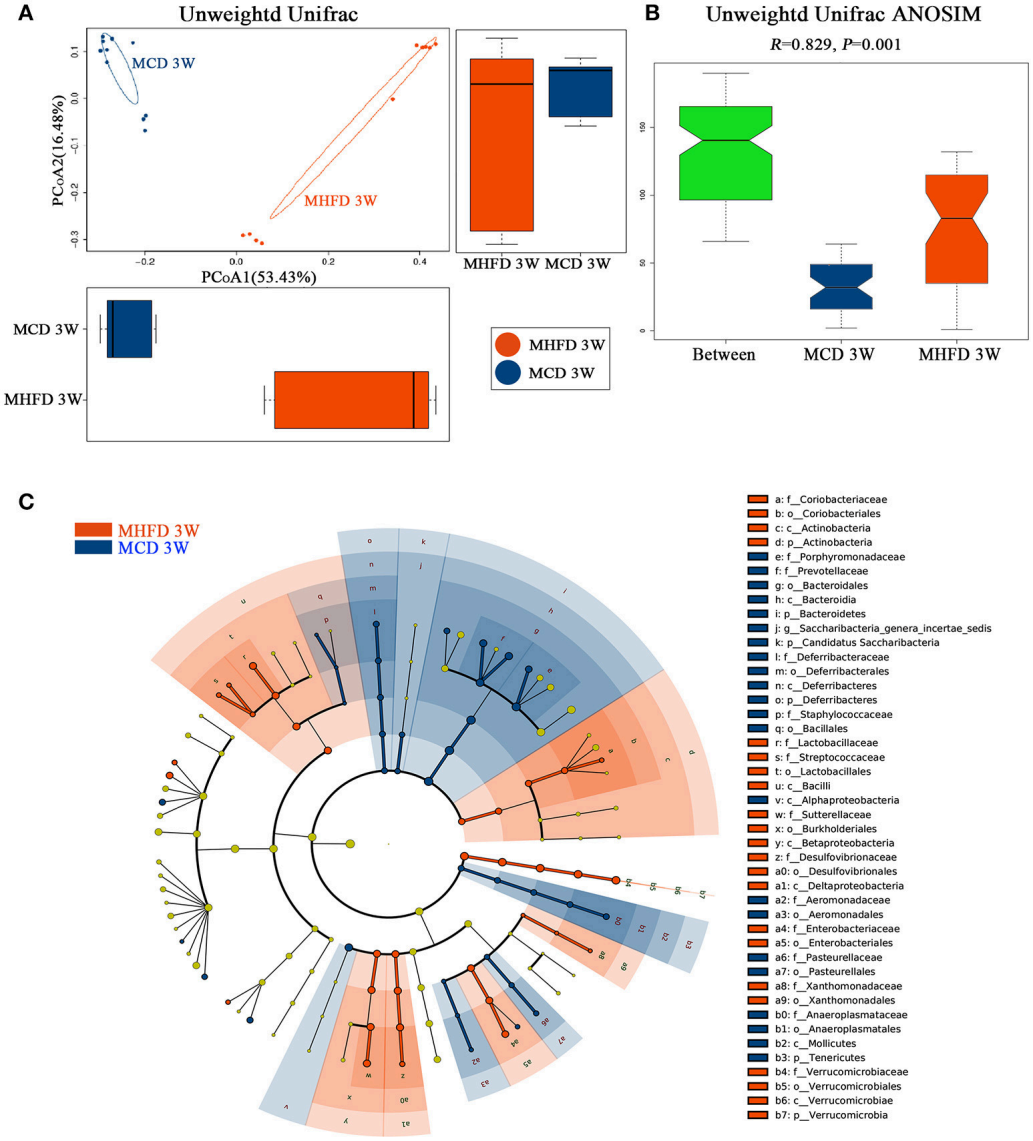
Maternal high fat diet altered the β diversity of gut microbiota and the key bacterial alterations in 3-week old offspring mice. Total fecal bacteria from each 3-week old offspring mice were detected by 16S rRNA sequencing. Beta diversity **(A)**, Unweighted Unifrac ANOSIM analysis between two groups **(B)** and differentially abundant species at the phylum, class, order, family, and genus level in Cladogram generated by LEfSe analysis **(C)** was shown. MHFD, maternal high fat diet. MCD, maternal control diet. In **(A–C)**, *n* = 10 in each group.

Then we performed principal coordinate analysis (PCoA) on unweighted UniFrac distances to characterize the differences among these samples. Significant coclustering of samples by maternal diet suggested that MHFD caused a pronounced influence on the microbial composition of 3-week old offspring (Figure 4A). To further quantify the differences in species diversity between two groups, Unweighted Unifrac Distance heatmap was projected. In agreement, MHFD pups clustered with MHFD pups and MCD pups clustered with MCD pups. ANOSIM analysis indicated that the differences between two groups were significant (Figure 4B). Differentially abundant species at the phylum, class, order, family, and genus level between MHFD and MCD group were examined by LDA EffectSize analysis (Figure 4C). Interestingly, the relative abundance of inflammation associated microbiota including *Peptostreptococcaceae* and *Streptococcus* (35) were higher in MHFD group. Meanwhile, *Lachnospiracea_incertae_sedis* and *Prevotellaceae*, which were generally linked to the production of butyric acid (36), were significantly decreased in MHFD group. The relative abundance of *Akkermansia*, a mucin-degrading bacterium (37), was increased in MHFD group, which may be associated with the disrupted mucosal barrier function in the 3-week old progeny.

Collectively, these data suggest that MHFD in early life could alter the microbiome in the offspring exposed to the maternal diet, which associated with a significant influence on the establishment and development of the neonatal microbiome.

### MHFD disrupted intestinal barrier function in 3-week old offspring mice

**Figure 5:**
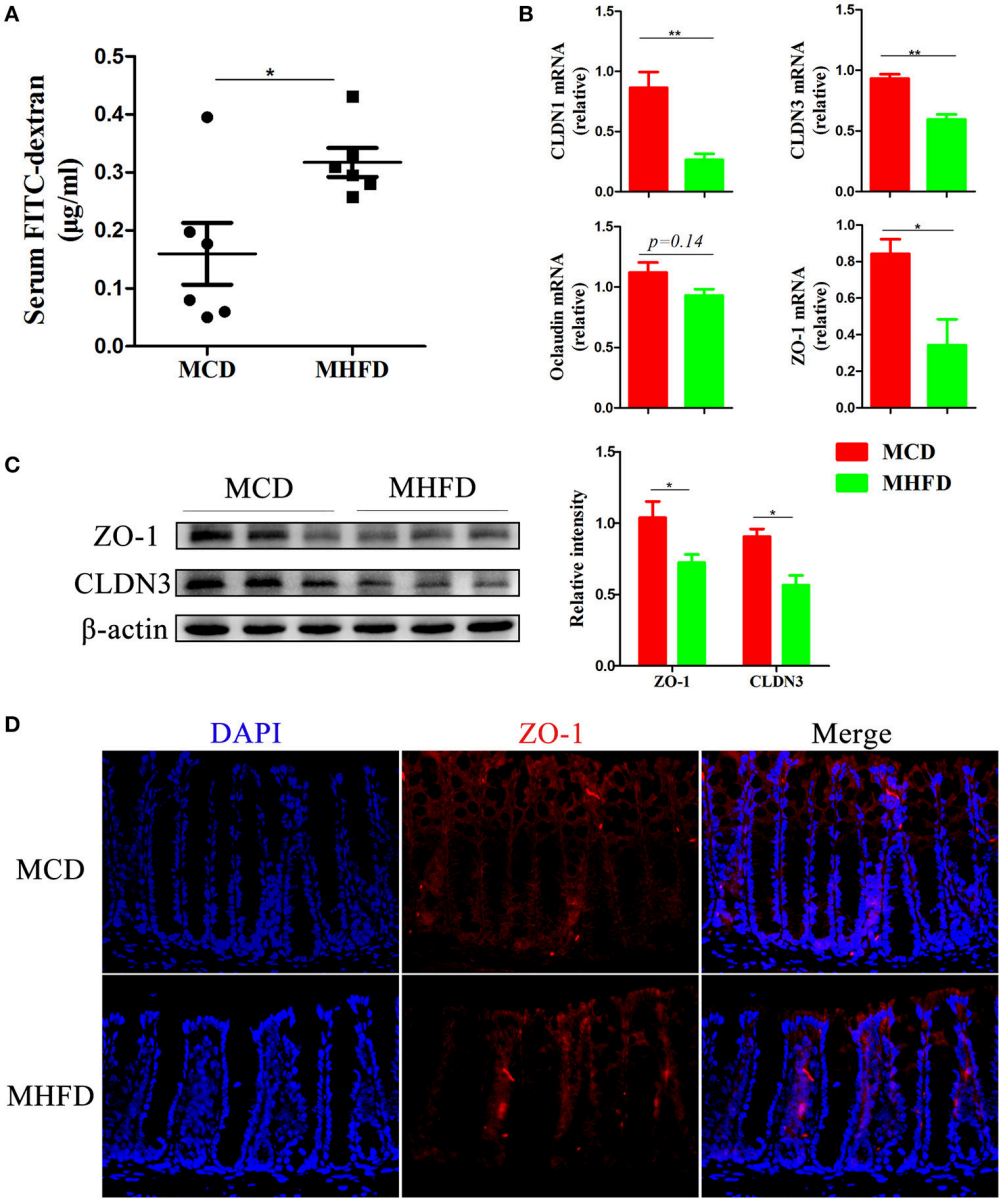
Maternal high fat diet disrupted intestinal barrier function in 3-week old offspring mice. Barrier formation was detected using the *in vivo* FITC-dextran assay. The FITC-dextran level in serum is shown **(A)**. Total RNA was extracted from the colonic tissues for real-time PCR analysis. The relative expression of CLDN 1, 3, Occludin, and ZO-1 was shown **(B)**. Protein levels of ZO-1 and CLDN3 in the colonic tissues from 3-week old offspring mice were detected by western blotting and the relative intensity was quantified **(C)**. The membrane localization of ZO-1 was assessed by immunostaining and visualized by fluorescence microscopy (red staining; 400 ×), nuclei were stained with DAPI (blue staining) and the red regions indicated ZO-1 **(D)**. MHFD, maternal high fat diet. MCD, maternal control diet. FITC-dextran, fluorescein isothiocyanate conjugated-dextran. In **(A–D)**, *n* = 6 in each group. ^*^p < 0.05, ^**^p < 0.01.

Recent evidence demonstrates that defects in the intestinal barrier, consisting of the external anatomic barrier and immunological barrier, play a major role in gastrointestinal disorders such as IBD (38). Here we investigated the effect of MHFD on the intestinal barrier function in 3-week old progeny. For the external anatomic barrier, fluorescein isothiocyanate conjugated-dextran (FITC-D) method was applied to detect the intestinal permeability. The serum level of FITC-D in MHFD group was significantly higher, suggesting that the intestinal permeability was increased and the integrity of tight junction was disrupted compared with MCD group (Figure 5A). Subsequently, tight junction proteins were measured. The expression of Claudin (CLDN) 1, CLDN3, and ZO-1 were significantly suppressed in MHFD group, and Occludin was also decreased although there was no significant difference between two groups (Figure 5B). The protein levels of cell-cell contact markers ZO-1 and CLDN3 were then detected by western blotting. As expected, our results showed that the protein levels of CLDN3 and ZO-1 were significantly decreased in MHFD group (Figure 5C). Besides, immunostaining of ZO-1 suggested that MHFD altered membrane localization of ZO-1 in the colon (Figure 5D).

**Figure 6:**
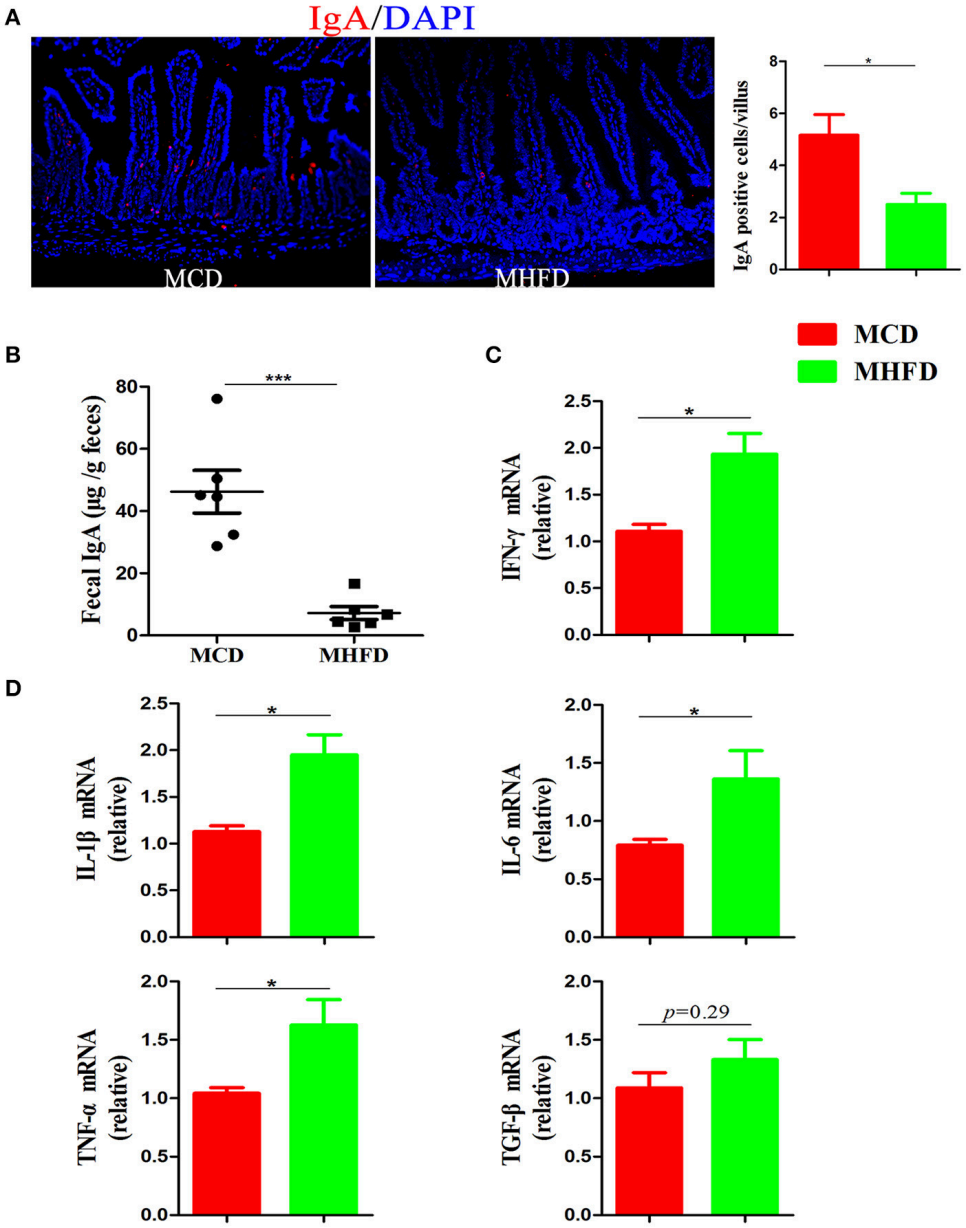
Maternal high fat diet decreased sIgA in the small intestine and induced colonic low-grade inflammation in 3-week old offspring mice. IgA in the small intestinal tissues from 3-week old offspring mice was assessed by immunostaining and visualized by fluorescence microscopy (red staining; 200 ×), IgA positive cells in 300 villi were counted **(A)**. Fecal sIgA levels were determined by ELISA **(B)**. Total RNA was extracted from the colonic tissues and prepared for real-time PCR analysis. The relative expression of inflammatory cytokines including IFN-γ **(C)**, IL-1β, IL-6, TNF-α, and TGF-β **(D)** was shown. MHFD, maternal high fat diet. MCD, maternal control diet. In **(A–D)**, *n* = 6 in each group. ^*^p < 0.05, ^***^p < 0.001.

Immunoglobulin A (IgA) is a main antibody in the gut and plays an essential role in the maintenance of intestinal immunological homeostasis (39, 40). In our study, immunostaining of IgA showed MHFD significantly reduced the number of IgA expressing cells in the small intestine (Figure 6A). Moreover, sIgA in feces were detected by ELISA and the results showed that sIgA levels were significantly decreased in MHFD group (Figure 6B). All these data illustrate that MHFD disrupted intestinal barrier function in 3-week old offspring.

### MHFD induced intestinal low-grade inflammation in 3-week old offspring mice

Although Xue et al. have reported that maternal obesity could induce gut inflammation in 16-week old non-obese diabetic offspring mice (41), the intestinal inflammatory condition of 3-week old progeny exposed to MHFD has not been investigated. In this study, H&E staining showed no apparent microscopic inflammation in both groups. The relative expression levels of inflammatory cytokines including IL-1β, IL-6, IFN-γ, and TNF-α mRNA were significantly higher in MHFD group (Figures 6C,D), while TGF-β did not reach significance (Figure 6D). Therefore, exposure to MHFD in early life can trigger intestinal chronic low-grade inflammation in offspring.

### MHFD-induced dysbiosis was not recovered in 8-week old offspring mice

**Figure 7:**
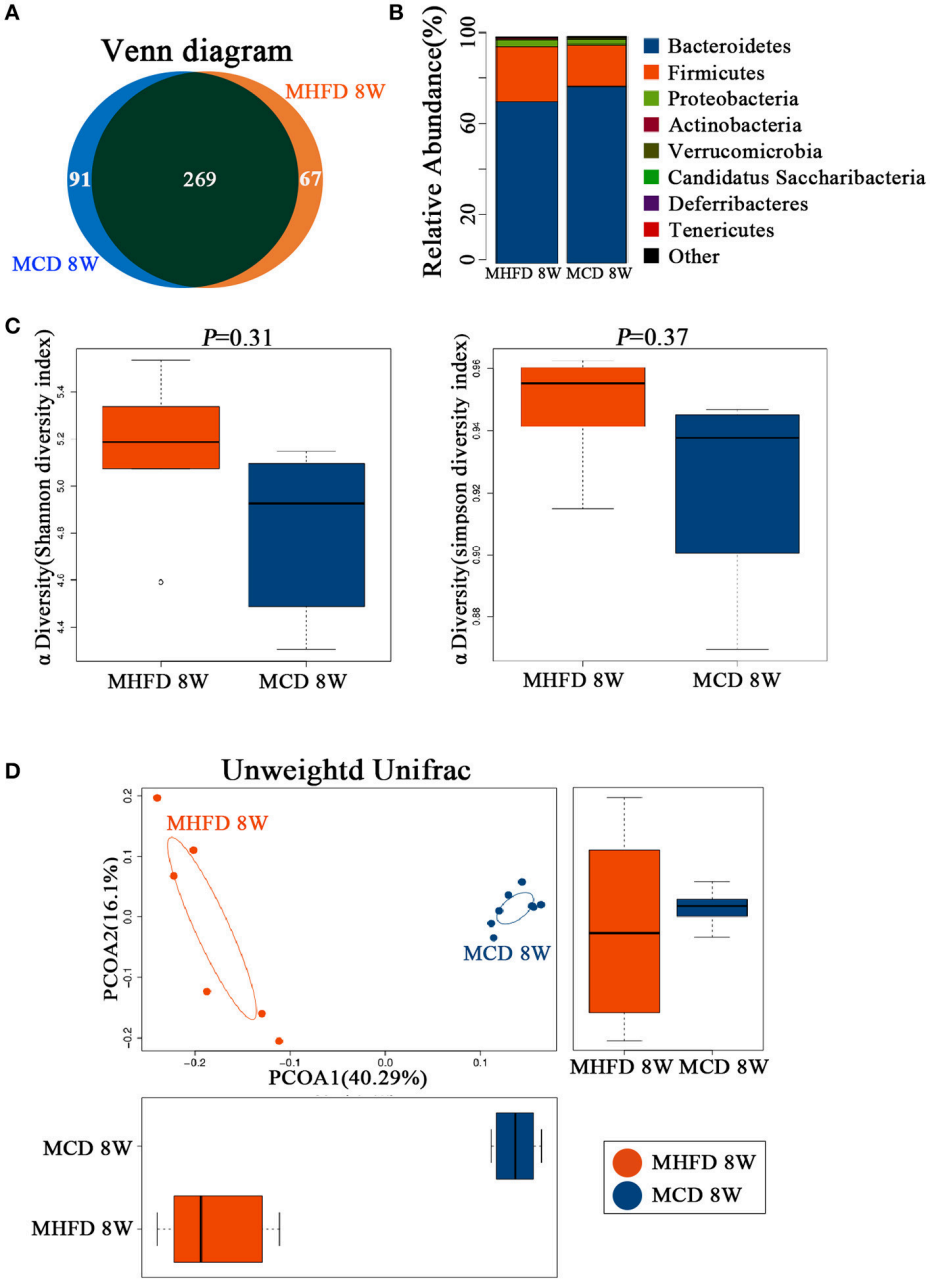
Maternal high fat diet altered the composition and diversity of gut microbiota in 8-week old offspring mice. Mice were treated as described in Figure 1A and total fecal bacteria from each 8-week old offspring mice were detected by 16S rRNA sequencing. Venn diagram **(A)**, the relative abundance of bacterial taxa at the phylum level between two groups **(B)**, Shannon and Simpson diversity index **(C)** was shown. Beta diversity was measured by Unweighted UniFrac distance **(D)**. MHFD, maternal high fat diet. MCD, maternal control diet. In **(A–D)**, *n* = 6 in MHFD 8 w group, *n* = 8 in MCD 8 w group.

There is widespread agreement that MHFD could cause alterations in the microbiota of offspring (25, 26). However, few studies concentrated on whether a change in diet could reverse the effect of MHFD on the offspring microbiota. In our studies, 3-week old offspring in both groups were fed with a control diet until 8 weeks of age and the gut microbiota was analyzed subsequently. Venn diagram showed 336 OTUs in MHFD group and 360 OTUs in the MCD group, with 269 OTUs shared (Figure 7A). No significant difference was observed among the *Firmicutes, Bacteroidetes, Proteobacteria, Actinobacteria*, and *Verrucomicrobia* at the phylum level of microbial composition at 8 weeks of age (Figure 7B). The Shannon and Simpson diversity index indicated that the decreased alpha diversity induced by MHFD at 3 weeks of age was partly recovered after 5-week period consumption of the control diet (Figure 7C).

**Figure 8:**
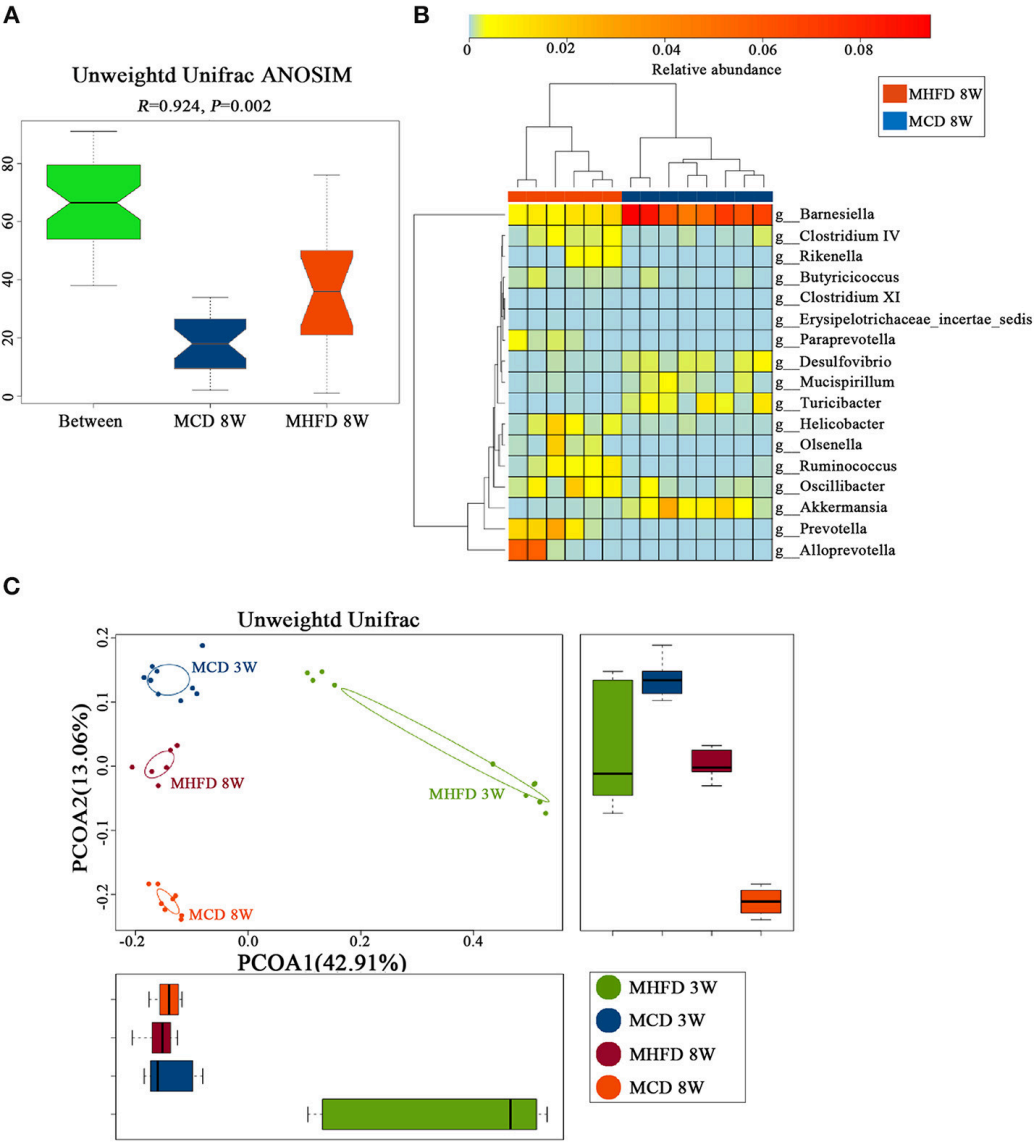
Maternal high fat diet-induced dysbiosis was not recovered in 8-week old offspring mice. Mice were treated as described in Figure 1A and total fecal bacteria from each 8-week old offspring mice were detected by 16S rRNA sequencing. Unweighted Unifrac ANOSIM analysis between two groups **(A)** and differentially abundant species at the genus level **(B)** was shown. The plots at 3 and 8 weeks of age were generated and measured by the Unweighted Unifrac PCoA **(C)**. MHFD, maternal high fat diet. MCD, maternal control diet. In **(A–D)**, *n* = 6 in MHFD 8w group, *n* = 8 in MCD 8w group. In, *n* = 10 in MHFD 3w and MCD 3w groups.

Afterwards, PCoA on unweighted UniFrac distances was generated. Our results revealed distinct clusters of microbiota composition between two groups at 8 weeks of age (Figure 7D). ANOSIM analysis revealed that the differences were significant (Figure 8A). Significantly different species at the genus level were represented in a heatmap (Figure 8B). Notably, inflammation associated microbiota including *Escherichia/Shigella, Helicobacter*, and *Oscillibacter* (7) was more abundant in MHFD group, whereas *Anaeroplasma* and *Lachnospiracea_incertae_sedis* (42), which can produce short-chain fatty acids (SCFA), were significantly decreased. In addition, the relative abundances of *Mucispirillum* and *Barnesiella*, which are perceived to benefit mucosa health (42, 43), were also remarkably decreased in MHFD group. Of note, PCoA at 3 and 8 weeks of age based on unweighted UniFrac distances indicated that the control diet after weaning indeed partly reversed the effect of MHFD on offspring microbiota (Figure 8C).

Together, our results reveal that MHFD significantly changed the composition of offspring gut microbiota at 8 weeks of age despite the bacterial diversity has been partly recovered.

### MHFD accelerated DSS-induced colitis in adult mice

**Figure 9:**
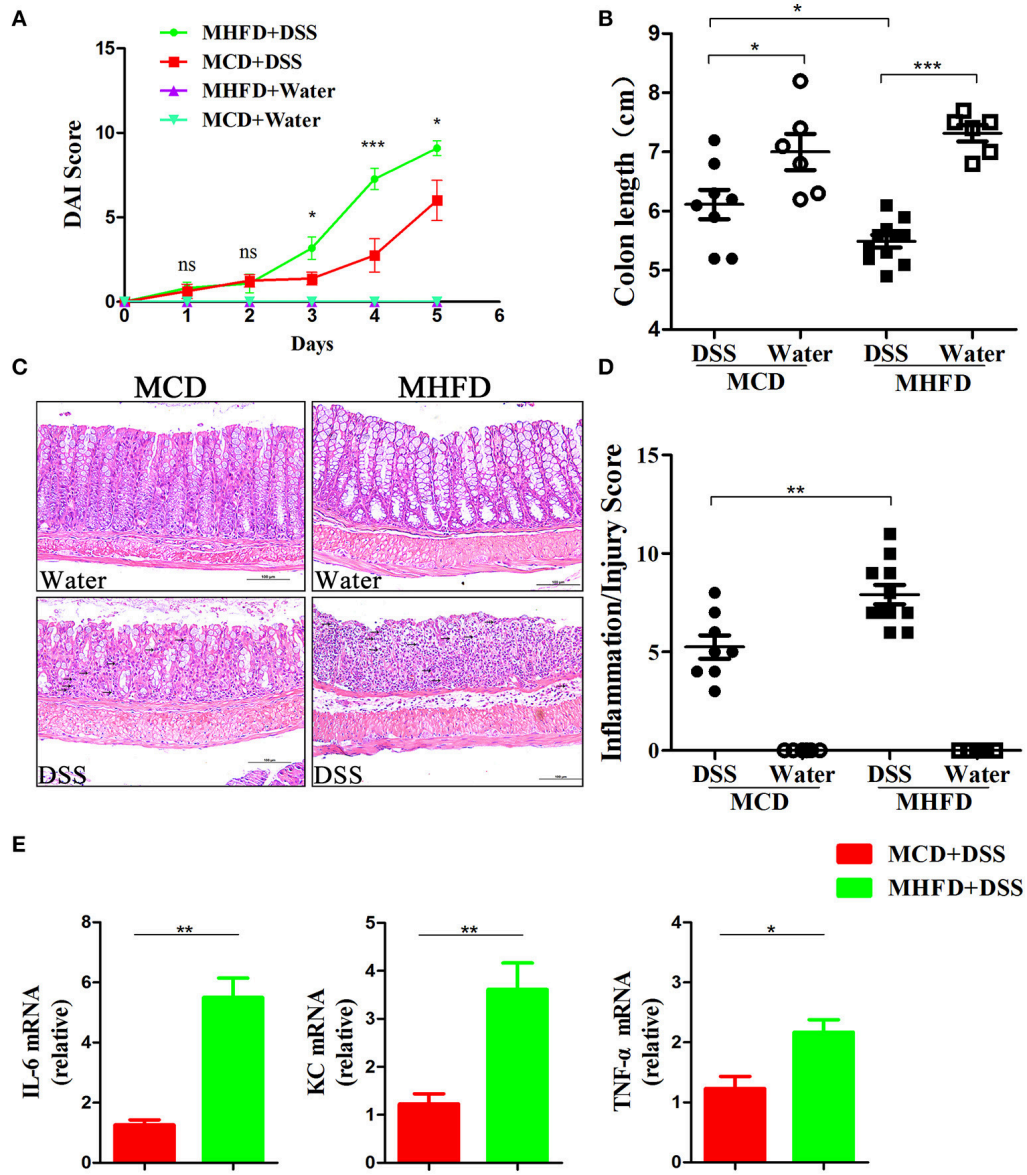
Maternal high fat diet exacerbated DSS-induced colitis in adult mice. Adult (8-week old) mice were treated with 2% DSS solution for 5 days. Disease activity index was recorded **(A)** and the length of the colon was measured **(B)**. Paraffin-embedded colon sections were stained with H&E for light microscopic assessment of epithelial damage and inflammation, immune cell infiltration was indicated by black arrows **(C)**. The inflammation/injury scores are shown **(D)**. The relative expression of IL-6, KC, and TNF-α was shown **(E)**. In **(A–D)**, *n* = 8 in MCD+DSS group, *n* = 11 in MHFD+DSS group, *n* = 6 in MCD+water and MHFD+water groups. In **(E)**, *n* = 6 in each group. MHFD, maternal high fat diet. MCD, maternal control diet. DSS, dextran sulfate sodium. DAI, disease activity index. Scale bar: 100 μm. ^*^p < 0.05, ^**^p < 0.01, ^***^p < 0.001.

Previous studies have demonstrated that alteration of the microbiota and chronic inflammation during development potentially lead to adverse outcomes in adulthood (17, 44, 45). Here we explored whether MHFD in early life increases the risk of colitis in adulthood. The offspring of either group was fed with a control diet until 8 weeks of age. They were then treated with 2% DSS for 5 days. DAI score was significantly higher in MHFD group at days 3, 4, and 5 after DSS treatment, and significant shortening of colon length was observed consistently compared with MCD group (Figures 9A,B). Moreover, no gross ulcerations were observed in both groups. The enhancement of colitis in MHFD group was confirmed by H&E staining. The histological inflammatory changes were performed as increased neutrophil and lymphocyte infiltrations and extended injury of intact crypt structures and surface epithelium (Figure 9C). Additionally, the inflammatory/injury score in the MHFD group (7.91 ± 1.6) was significantly higher than MCD group (5.25 ± 1.7, *p* < 0.01) (Figure 9D). Furthermore, we examined the expression of pro-inflammatory cytokines in DSS-treated mice. Elevated expression of IL-6, KC, and TNF-α was observed in MHFD group, as compared with MCD group (Figure 9E). Collectively, these observations indicate that MHFD significantly exacerbated the susceptibility of offspring to colitis in adulthood.

## Discussion

The incidence of IBD is rising and has become a great challenge worldwide but the pathogenesis has not been completely explained (46). Recently, environmental factors especially early life events are considered to be associated with the development of IBD (16). It has been demonstrated that MHFD in early life can alter the offspring microbiome and have a longstanding impact on offspring (47–51). In the present study, we investigated the effect of MHFD on intestinal development, gut microbiome, intestinal inflammation, and barrier function of the offspring. Our results demonstrated that MHFD in early life could alter intestinal development, tight junction formation, intestinal microbial composition, and inflammatory molecules of offspring mice and increase the susceptibility to DSS-induced colitis in adulthood. This result may partly explain why the MHFD in early life has been proposed to be an environmental risk factor in IBD pathogenesis.

It is commonly accepted that maternal obesity is associated with an increased risk of childhood overweight or obesity (52, 53), which may contribute to metabolic disorders and IBD later in life (54, 55). However, the relationship between MHFD during pregnancy and lactation and offspring obesity remains obscure (56–58). As reported by King et al. increased weight gain in offspring exposed to MHFD is only observed before weaning (59). In our study, we found that pups in MHFD group exhibited an increased body weight from birth to 8 weeks, but the significant difference was only observed at 2, 3, and 4 postnatal weeks. Moreover, according to a recent population-based cohort study, increased childhood BMI is only associated with early-onset CD (before 30 years of age) but not with UC (60). All these suggested that the enhanced susceptibility of offspring to colitis in adulthood were independent of body weight gain in childhood.

Maternal factors are undoubtedly essential to modify the intestinal development of offspring (61, 62). Previous studies have already shown that early supplementation with some probiotics or lactoferrin could promote intestinal proliferation and differentiation (22, 63). Additionally, reports have also found that maternal high-energy diet, rich in polyunsaturated fatty acids, can promote intestinal development in offspring sows (64). In contrast, the effect of MHFD (high in saturated fatty acids) on the intestinal development of offspring has not been clearly described. A recent study shows that maternal exposure to a high fat diet significantly alters the expression of genes involved in modulating normal development, function, and immune response of the gut by interaction with intestinal microbiota in 2-week old offspring (26). Moreover, maternal dysbiosis and decreased antioxidant defense capacity induced by a high fat diet reportedly results in an aberrant intrauterine environment, which could lead to poor liver and hippocampus development of the fetus (65–67). Data from our study showed that MHFD negatively impacts the intestinal development of offspring at the early stage, whereas similar findings were not found in adulthood.

Several studies have provided evidence that MHFD could alter the microbiota of offspring in both human and animals at the early stage (26, 48–50). In our study, although inter-animal variance does exist in MHFD group, we found that MHFD significantly changes the composition and diversity of 3-week old offspring characterized with increased *Firmicutes*/*Bacteroidetes* ratio, decreased alpha-diversity, and reduction of *Butyricicoccus, Bacteroides*, and *Alloprevotella* at the genus levels. Given fecal samples of MHFD group were collected from 5 cages randomly, maternal transmission, genetic drift, and cage effects which play a critical role in influencing microbiota composition (68) may explain why the variance was displayed in MHFD group. Along this line, our data demonstrate that MHFD in early life can alter the composition and diversity of offspring microbiota at early stage, even though an inter-animal variance was displayed within the groups. However, further works are required to get better results.

Certain studies have demonstrated that early colonization of gut microbiota in prenatal and infant period is critical for the development of the immune system (69–71). Disturbance of the early colonization may increase the risk of autoimmune diseases (72, 73). It has been proposed that decreased bacterial exposure could influence the maturation of inducible regulatory T cells (iTregs) induction, which may be responsible for the decreased secretion of anti-inflammatory cytokines (74). Moreover, decreased abundance of *Butyricicoccus, Prevotellaceae*, and *Lachnospiracea_incertae_sedis*, which are regarded as SCFA producing bacteria, were also linked to the reduction of iTregs and activation of proinflammatory cells (71, 75). Although Val-Laillet et al. and Steegenga et al. have demonstrated that maternal high fat diet can alter microbiota composition of offspring (26, 50), the correlation between the alterations and intestinal inflammation or barrier function is limited. We observed MHFD can increase the level of inflammatory cytokines and trigger chronic low-grade intestinal inflammation in offspring at 3 weeks of age. Of note is that dysbiosis or immune response was not observed when offspring exposed to MHFD and MCD was cohoused together (48), suggesting that the key determinant factor of MHFD on offspring immune system is attributed to the dysbiosis.

Animal studies have shown that dysbiosis induced by high fat diet is implicated in the decrease of SCFA and affects intestinal barrier function subsequently in adult mice (76–78). Xue et al. have also reported that maternal obesity impairs gut epithelial barrier function in non-obese diabetic offspring mice at 16 weeks of age (41), despite it is difficult to accurately determine the effect of maternal obesity. However, the intestinal barrier function at the early stage is less investigated. In this study, we observed a reduction of goblet cells and MUC2 positive cells in the colon, together with reduced cell-cell contact mRNA levels and elevated FITC serum level. These findings suggest that MHFD significantly disrupt the barrier function of offspring at 3 weeks of age. Further, in line with the microbiota difference, the relative abundance of *Akkermansia* was dramatically increased in MHFD group. Given *Akkermansia* is a mucin-degrading bacteria, this may partly contribute to the decrease of the mucin secreted by the goblet cells (36). However, the underlying mechanisms of the microbial modulation and pro-inflammatory effect needs further study.

Although accumulating evidence points to a critical role for intestinal dysbiosis in IBD, the relationship between microbiota and IBD remains largely unresolved. Several studies have demonstrated perturbed gut microbiota induced by early life antibiotics exposure has been proposed as the underlying mechanism of immune-related diseases, including IBD and type 1 diabetes (79, 80), but how the perturbations link to the diseases later in life are remarkably complex. Regardless of the methodology used, a recent study also reported the use of maternal high-fat diet could enhance DSS-induced colitis (27). However, underlying mechanisms especially the association between microbiota and colitis were not investigated. In the present study, colitis was greatly enhanced in MHFD group. The relationships between the microbiota and colitis are undeniable. We found that microbial difference between two groups still exists despite the altered alpha diversity and microbial composition at the phylum level induced by MHFD partly recovered at 8 weeks of age. The relative abundance of *Escherichia/Shigella, Helicobacter*, and *Oscillibacter*, which are considered as possible inflammation promoting agents, were significantly increased prior to DSS treatment in MHFD group. Meanwhile, we also observed that the relative abundances of *Mucispirillum* and *Barnesiella*, which are beneficial to mucosa health, were remarkably decreased in MHFD group. As disturbances of mucosal barrier function are involved in the pathogenesis of IBD, these alterations might contribute to increase the risk of colitis. Taken together, these findings demonstrate MHFD can alter the microbial composition of adult offspring and thus facilitate the susceptibility of colitis. Germ-free mice experiments will be further needed to validate the causality.

In summary, although a small portion of our results has been manifested in previous publications, this study first characterizes that MHFD in early life could change intestinal development and cell proliferation and alter the composition and diversity of intestinal microbiota, as well as induce low-grade inflammation and disrupt mucosal barrier function in 3-week old offspring collectively. Moreover, the microbial analysis in adulthood indicate that although bacterial diversity recovered after the control diet, perturbations of microbial composition still exist; which likely contributed to exacerbating the susceptibility of experimental colitis in adulthood. These observations support the hypothesis that MHFD in early life may be a potential risk factor for IBD. As high fat diet is common in pregnant women especially in developing countries, our findings might contribute to a better understanding of the increasing incidence of IBD. Furthermore, supplementation with probiotics or avoiding high fat diet in early life might be novel options for prevention of IBD.

## Methods and materials

### Ethical approval statement

All experimental procedures were performed according to the guidelines of the Institutional Animal Care and Use Committee at Tianjin Medical University and followed the International Association of Veterinary Editors guidelines for the Care and Use of Laboratory Animal. The animal use protocol listed below has been reviewed and approved by the Animal Ethical and Welfare Committee of Tianjin Medical University, Approval No. TMUaMEC 2016011.

### Mice and diets

Adult C57BL/6 female mice (8 weeks of age) from the same litter were purchased from the Institute of Laboratory Animal Science, Chinese Academy of Medical Sciences & Peking Union Medical College, and were placed in the light- and temperature-controlled facility under the specific pathogen-free circumstance. Subsequently, they were mated with C57BL/6 males on control diet and checked daily. After pregnancy was confirmed by postcopulatory plugs, female mice in MHFD group were switched to the high fat diet. Pregnant mice were housed together until 1 week before delivery, and then they were housed singly in the separated cages. Dams had free access to drinking water. Throughout the period of gestation (3 weeks) and lactation (3 weeks), MHFD group dams consumed the high fat diet (HFD, 5.24 kcal/g; 60 E% fat, 20 E% protein, 20 E% carbohydrate; H10060, Research Diets, Peking, China) whereas MCD (maternal control diet) group dams consumed the control diet (CD, 3.85 kcal/g; 10 E% fat, 20 E% protein, 70 E% carbohydrate; H10010, Research Diets, Peking, China) constantly. Afterwards, dams delivered vaginally and the body weight of pups was monitored at birth and weekly until 8 weeks. Pups were weaned at 3 weeks of age, and separated from the dams and housed 5 per cage and received regular drinking water and standard rodent chow (3.85 kcal/g; 10 E% fat, 20 E% protein, 70 E% carbohydrate; H10010, Research Diets, Peking, China) *ad libitum* till 8 weeks (Figure 1A).

### Tissue collection

Mice were sacrificed under anesthesia at 3 and 8 weeks of age and then the whole intestine and colon were excised instantly. Small intestinal and colonic tissues were cleaned by ice-cold phosphate-buffered saline (PBS) solution. The tissues were then embedded into Swiss rolls in paraffin followed by hematoxylin and eosin (H&E) staining for evaluation of villi length and crypts depth or snap-frozen rapidly, and stored at −80°C for later analysis of mRNA and protein expression.

### Intestinal permeability assay

The intestinal permeability of 3-week old mice was determined by the FITC-D method. FITC-D (4000 MW, Sigma-Aldrich) dissolved in normal saline infusion (50 mg/mL) and was administrated to the mice by gavage at 6 mg/10 g body weight. Whole blood was collected 4 h after FITC-D administration using heparinized microhematocrit capillary tubes via eye bleed. Sera was extracted from the blood by centrifuging at 4°C for 10 min at 2,000 rpm. Fluorescence intensity analysis was carried out using a plate reader. The concentration of FITC-D of each mouse was detected based on the FITC-D standard curve.

### Intestinal microbiota analysis

The 16S rRNA gene sequencing procedure was performed by the Realbio Genomics Institute (Shanghai, China). Total fecal bacteria DNA extractions were acquired from cecal specimens of each 3-week old and 8-week old offspring by QIAamp ® Fast DNA Stool Mini Kit (QIAamp, Germany). The microbial 16S V3-V4 region was amplified with indexes and adaptors-linked universal primers (341F: ACTCCTACGGGAGGCAGCAG, 806R: GGACTACHVGGGTWTCTAAT). PCR was performed using KAPA HiFi Hotstart PCR kit high fidelity enzyme in triplicate. Amplicon libraries were quantified by Qubit 2.0 Fluorometer (Thermo Fisher Scientific, Waltham, US) and then sequenced on Illumina HiSeq platform (Illumina, San Diego, US) for paired-end reads of 250 bp. After discarding the singletons and removing chimeras, tags were clustered into operational taxonomic units (OTUs) using USEARCH (v7.0.1090) at 97% similarity. Afterwards, a representative sequence of each OTU was subjected to the taxonomy-based analysis using the RDP database. Heatmap was created using R. Cluster analysis. Alpha diversity (Shannon, Simpson) and beta diversity were analyzed using QIIME. The relative abundance of bacteria was expressed as the percentage.

### Realtime-PCR analysis

Total RNA was extracted using the RNeasy mini kit (Qiagen, Carlsbad, CA, USA) followed by cDNA reverse transcription using the TIANScript RT Kit (TIANGEN, Inc. Beijing, China) according to the manufacturer's protocol. Realtime-PCR analysis was performed using Taqman Gene Expression Master Mix and primes (GENEWIZ, Inc. Beijing, China). The Oligonucleotide primers for target genes were listed in Table 1. Glyceraldehyde-3-phosphate dehydrogenase (GAPDH) was employed as an endogenous control. The relative mRNA expression levels of the target gene were evaluated by calculating the fold-changes normalized to the GAPDH for each sample using 2^−ΔΔ*Ct*^ method. All cDNA samples were analyzed in triplicate.

**Table 1:** The Oligonucleotide primers used in Realtime-PCR analysis.

**Murine gene**	**Primer sequences (5^′^- 3^′^)**
GAPDH	Forward primer: TGTGTCCGTCGTGGATCTGA Reverse primer: CCTGCTTCACCACCTTCTTGA
ZO-1	Forward primer: GGGCCATCTCAACTCCTGTA Reverse primer: AGAAGGGCTGACGGGTAAAT
Occludin	Forward primer: ACTATGCGGAAAGAGTTGACAG Reverse primer: GTCATCCACACTCAAGGTCAG
Claudin 1	Forward primer: GAATTCTATGACCCCTTGACCC Reverse primer: TGGTGTTGGGTAAGAGGTTG
Claudin3	Forward primer: CCTGTGGATGAACTGCGTG Reverse primer: GTAGTCCTTGCGGTCGTAG
IL-1β	Forward primer: GTGGCTGTGGAGAAGCTGTG Reverse primer: GAAGGTCCACGGGAAAGACAC
IL-6	Forward primer: CCAGTTGCCTTCTTGGGACT Reverse primer: GGTCTGTTGGGAGTGGTATCC
TNF-α	Forward primer: ACTCCAGGCGGTGCCTATG Reverse primer: GAGCGTGGTGGCCCCT
MUC2	Forward primer: TCGCCCAAGTCGACACTCA Reverse primer: GCAAATAGCCATAGTACAGTTACACAGC
TGF-β	Forward primer: GCTGAACCAAGGAGACGGAAT Reverse primer: GCTGATCCCGTTGATTTCCA
IFN-γ	Forward primer: GCATCTTGGCTTTGCAGCT Reverse primer: CCTTTTTCGCCTTGCTGTTG
KC	Forward primer: AACCGAAGTCATAGCCACAC Reverse primer: CAGACGGTGCCATCAGAG

### Western blotting

The colonic tissues were dissolved in RIPA buffer with protease inhibitors (Solarbio, Beijing, China). After homogenization, the protein concentrations were determined by Bicinchoninic acid protein assay (Thermo Scientific Inc). Proteins were separated using SDS-polyacrylamide gel electrophoresis system and then blotted onto a polyvinylidene fluoride (PVDF) membrane (Invitrogen, Carlsbad, CA, USA). Afterwards, the primary anti-CLDN3 (rabbit, antimouse, Cell Signaling Technology), anti-ZO-1 (rabbit, antimouse, Cell Signaling Technology) and anti-β-actin (rabbit, antimouse, Cell Signaling Technology) antibody were applied; anti-β-actin antibody was employed as the loading control. After incubated with horseradish peroxidase (HRP)–conjugated secondary antibodies (Cell Signaling Technology), the chemiluminescent signal was detected. The intensity of the band was determined by image processor program (Image J).

### Measurement of fecal sigA

Fecal samples were homogenized by placing in homogenization buffer (phosphate buffer saline (PBS) containing 0.05% Tween 20 and Protease Inhibitor Cocktail) for 15 min at 5,000 revolutions per minute. Subsequently, homogenates were briefly centrifuged and the supernatants were frozen at negative 20°C for subsequent assays. Standards and fecal samples were added to the appropriate well of 96-well polystyrene anti-mouse IgA antibody pre-coated Microtiter Plate (Sigma-Aldrich) according to manufacturer's instructions. All samples were tested in duplicate and the mean optical density (OD) was calculated.

### Periodic acid schiff (PAS) staining

Deparaffinized colonic sections were incubated with 1% Periodic acid solution (Sigma-Aldrich) for 10 min followed by staining in Schiff reagent (Sigma-Aldrich) for 40 min. Afterwards, the PAS-stained sections were counterstained with hematoxylin for 2–5 min. PBS solution was used as washing buffer for rinsing in every step.

### Histology and immunohistochemistry

The intestinal tissues were fixed in 4% formalin solution, embedded in paraffin, and cut into 4-μm slices. After deparaffinization and hydration, H&E staining was performed for evaluation of intestinal development and inflammation. The intestinal development was assessed by measurement of villus/crypt length. Three hundred well-orientated villi/crypts randomly chosen from × 200 images was observed and measured under light microscope SZX16 (Olympus, Japan) for each mouse.

For immunohistochemistry, deparaffinized sections were incubated with primary antibodies rabbit monoclonal anti-Ki67 (ab16667, Abcam, Cambridge, MA, USA) and rabbit anti-MUC2 (Santa Cruz Biotechnology, Inc) overnight at 4°C. After washing in PBS, sections were further incubated with the biotinylated anti-rabbit secondary antibody (Santa Cruz Biotechnology, Inc) for 30 min followed by counterstaining with 3, 3′-diaminobenzidine. The expression of Ki67 and MUC2 was detected by counting the absolute number of positively stained cells. Five random areas from × 400 images were performed under light microscope DM5000 B (Leika, Germany) in at least 100 villi or crypts for each mouse.

### Immunofluorescent staining

The formalin-fixed small intestine and colon tissues were embedded in paraffin and cut into 4 μm sections. After deparaffinization and hydration, sections were incubated 15 min with Antigen Unmasking Solution (Vector Laboratories, Inc. Burlingame, CA, USA) for antigen retrieval. Subsequently, 5% BSA was used to block non-specific binding. Then the small intestinal sections were incubated with specific primary anti-IgA antibody (ab223410, Abcam, Cambridge, MA, USA), and colonic sections were incubated with the specific primary anti-ZO-1 antibody (ab96587, Abcam, Cambridge, MA, USA) overnight at 4°C. Subsequently, the sections were washed with 1 × PBS for 5 min three times and incubated with fluorochrome-conjugated secondary antibodies IgG H&L fluorescent secondary antibody (Santa Cruz Biotechnology, Inc) and ALEXA FLUOR 488(FITC) secondary antibody (Santa Cruz Biotechnology, Inc) at room temperature in the dark for 60 min. DAPI (4, 6-diamidino-2-phenylindole) was lastly applied to dye the nucleus. Fluorescence photographs were obtained under fluorescence microscope DM5000 B (Leika, Germany). FITC and DAPI images were taken from a unified area. Quantitative analysis of immunofluorescence staining for IgA was performed (81). IgA positive cells in 300 villi were counted.

### Dextran sulfate sodium (DSS) induced colitis

DSS (36–50kDa, MP Biomedicals) was dissolved in drinking water and 8-week old mice were fed with 2% DSS solution for 5 days. Mice in both groups were given tap water as a control for DSS treatment. The DSS solutions were changed every day to maintain fresh until the end of the experiment. Disease activity index (DAI) scoring system which consists of the changes in animal weight loss, the presence of occult or gross blood per rectum and stool consistency was applied to determine the severity of colitis. Mice were sacrificed and the entire colon was quickly excised since the colon length was measured as a marker for inflammation. Colonic tissue was either stored at −80°C until used for inflammation cytokines profiles analysis or fixed in 10% buffered formalin and embedded in paraffin for H&E staining. Histological damage was assessed as a combined score of the degree of inflammation (scale of 0–3), the percentage of area involved by inflammation (0–4), crypt damage (0–4), and depth of inflammation (0–3) in a blinded way by a pathologist (YJZ).

### Statistical analysis

Data were presented as the mean ± SEM. The statistical significance of differences was assayed by one-way ANOVA in multiple groups, and *t*-tests for paired samples using SPSS 22.0 (SPSS, Chicago, IL, USA). All the differences were considered as statistically significant at *p* < 0.05.

## Author contributions

RX, HC, and BW designed the study. RX, YS, GJ, JW, ZG, XL, and TL performed the experiments. RX, YS, SH, XC, and YZ analyzed the results. RX and HC wrote the paper.

### Conflict of interest statement

The authors declare that the research was conducted in the absence of any commercial or financial relationships that could be construed as a potential conflict of interest.

## Supplementary material

Supplementary Figure 1 Maternal high fat diet decreased the MUC2 mRNA expression in 3-week old offspring mice. Total RNA was extracted from the colonic tissues of 3-week old offspring mice for real-time PCR analysis. The relative expression of MUC2 mRNA was shown. *n* = 6 in each group. MHFD, maternal high fat diet. MCD, maternal control diet. ^**^*p* < 0.01.
