# Supplementary figures and images for: Maternal High Fat Diet Alters Gut Microbiota of Offspring and Exacerbates DSS-Induced Colitis in Adulthood
Source: Front Immunol. 2018 Nov 13;9:2608. doi: 10.3389/fimmu.2018.02608 (PMC6243010; doi:10.3389/fimmu.2018.02608)

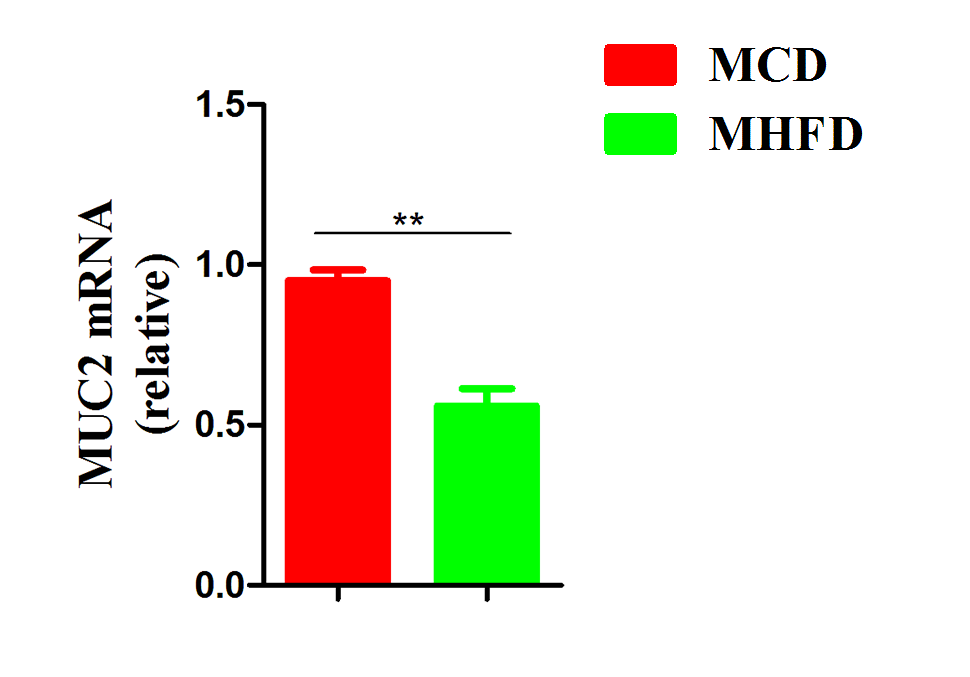

Supplement: Supplementary Figure 1 — Maternal high fat diet decreased the MUC2 mRNA expression in 3-week old offspring mice. Total RNA was extracted from the colonic tissues of 3-week old offspring mice for real-time PCR analysis. The relative expression of MUC2 mRNA was shown. n = 6 in each group. MHFD, maternal high fat diet. MCD, maternal control diet. **p < 0.01. [file Image_1.tif]
